# Effect of handling and crowding on the susceptibility of Atlantic salmon (*Salmo salar* L.) to *Lepeophtheirus salmonis* (Krøyer) copepodids

**DOI:** 10.1111/jfd.13286

**Published:** 2020-10-28

**Authors:** Cyril Delfosse, Patrick Pageat, Céline Lafont‐Lecuelle, Pietro Asproni, Camille Chabaud, Alessandro Cozzi, Cécile Bienboire‐Frosini

**Affiliations:** ^1^ Research Institute in Semiochemistry and Applied Ethology (IRSEA) Apt France; ^2^ IRSEA‐ARC Daugstad Norway

**Keywords:** cortisol, handling stress, host‐parasite interactions, parasite attachment, salmon louse, stocking density

## Abstract

*Lepeophtheirus salmonis* is an ectoparasite causing economic concerns in Atlantic salmon farming. Salmon lice infestation management methods can be stressful and impact fish welfare. This work investigated the stress effect on the attachment of *L. salmonis* copepodids to Atlantic salmon through two approaches: (a) handling by netting and air exposure (acute stress), and (b) crowding with restricted surface access in a tank (chronic stress). In the first experiment, we compared the number of attached *L. salmonis* and cortisol levels between a group of handled salmon and a control group. In the second experiment, a group of densely packed salmon was compared to a control group based on the number of attached copepodids, cortisol levels and neutrophil:lymphocyte ratios. Handled salmon showed significantly higher plasma cortisol levels (*p* < .001) and more attached copepodids (*p* = .01) than control salmon. Conversely, the cortisol level and number of attached copepodids were not significantly different between the densely packed and control salmon (*p* > .05). The neutrophil:lymphocyte ratio was significantly higher (*p* = .0014) in the densely packed salmon than in the control salmon. Handling salmon increased their risk of infestation by *L. salmonis*. This has implications for reinfestation rates following delousing treatments in commercial salmon aquaculture, which often involve crowding and handling salmon.

## 1 INTRODUCTION

Sea lice are crustacean caligid ectoparasites that feed on host marine fish tissues and cause major issues in marine aquaculture. Three lice species present the main concerns for salmon farming: *Lepeophtheirus salmonis* and *Caligus elongatus* in the Northern Hemisphere and *Caligus rogercresseyi* in the Southern Hemisphere. *L. salmonis* is naturally found on salmonids to which it is almost exclusive, hence is also referred to as the salmon louse. (Boxaspen, 2006; Johnson et al., 2004; Pike & Wadsworth, 1999; Tully & Nolan, 2002). The life cycle of *L. salmonis* contains eight stages: two free‐swimming stages (nauplius I and II), one infestation stage (copepodid) and five parasitic stages (chalimus I and II, pre‐adult I and II and adult) (Hamre et al., 2013). Regarding the infestation stage, copepodid attachment behaviour is triggered by a combination of visual, mechanosensory and chemosensory stimuli (Fields et al., 2018; Mordue & Birkett, 2009). Some of the semiochemicals released by the mucus of fish have already been demonstrated to be involved in host‐parasite interactions (Bailey et al., 2006; Delfosse & Pageat, 2014; Ingvarsdóttir et al., 2002; Mordue & Birkett, 2009; Núñez‐Acuña et al., 2018; Pageat & Delfosse, 2014; Pino‐Marambio et al., 2007).

In Atlantic salmon (*Salmo salar*) aquaculture in the Northern Hemisphere, *L. salmonis* negatively affects the welfare of farmed salmon, causing significant economic loss for the salmon aquaculture industry, resulting from damage to salmon as well as treatment costs (Brooker et al., 2018; Costello, 2009; Liu & Bjelland, 2014; Mordue & Birkett, 2009). In fact, sea lice infestations may cause a reduction in growth rates and increase mortality, morbidity and secondary infections in Atlantic salmon (Johnson et al., 2004; Pike & Wadsworth, 1999; Torrissen et al., 2013). Therefore, during the rearing process, sea lice infestations must be contained to a sustainable level by farmers (Abolofia et al., 2017).

Sea lice count is mandatory in Norway, and a fish farmer must report the amount of sea lice on the fish on a weekly basis to monitor the infestation level and to maintain it below legal thresholds (Abolofia et al., 2017; Marques et al., 2018; Norwegian Government, 2019). This scenario means that fish must be sampled in their cages by netting and maintained out of the water, air‐exposed, before anaesthesia and manual inspection. Furthermore, the management of salmon lice infestation requires delousing methods that can be stressful for the fish and jeopardize its welfare (Overton et al., 2018). Historically, chemical interventions through bathing treatments and medicinal feed have been extensively used (Aaen et al., 2015). Bath treatments are performed either by lining a sea cage with a tarpaulin and reducing the volume of water within the cage, or by pumping fish into a well‐boat, both resulting in densely packed salmon (Overton et al., 2018). Due to the development of lice resistance to chemical treatments, there is an increase in technological innovation development around the management of sea lice infestations (Aaen et al., 2015). Those include delousing treatments such as freshwater bathing, mechanical removal, thermal treatments and laser delousing (Overton et al., 2018). Mechanical and thermal delousing (using cold or warm water) typically involves some pumping and crowding prior to the treatments, which can themselves damage the skin and the mucus layer, leading to significant physical stress (Espmark et al., 2016; Gismervik et al., 2019; Overton et al., 2019).

Additionally, there is increasing interest in prevention methods (Barrett et al., 2020). Snorkel sea cages are currently presented as an interesting approach to diminish sea lice loads on farmed salmon, with no effect on fish growth and a welfare index based on fish visual appearance (Geitung et al., 2019; Oppedal et al., 2017; Stien et al., 2016). These submersible cages keep the salmon at depths where the sea lice are less abundant and are equipped with a tube or snorkel, impermeable to sea lice larvae, to provide surface access since salmon, as physostomes, need to reach the surface to refill their swim bladder with air. This technology may intensify temporary local fish crowding when fish use the snorkel and induce surface access limitation, especially in small snorkels, which might lead to negative welfare outcomes in salmon (Turnbull et al., 2005; Wright et al., 2017, 2018). Overall, salmon conditions (health and welfare) could be affected in many situations faced during salmon lice monitoring, delousing or prevention methods since handling and crowding occurs in almost all types of louse management (Ashley, 2007; Segner et al., 2012).

Handling and overcrowding have already been demonstrated to be stressful situations resulting in activation of the hypothalamus–pituitary–interrenal axis (i.e. cortisol or other related stress indicators variations; Erikson & Misimi, 2008; Liu et al., 2015; Merkin et al., 2010). However, the consequences of these procedures on salmon‐sea lice interactions have never been studied. In fact, among the different factors involved in the copepodids attachment success on salmon, sea lice management procedures could themselves modify salmon susceptibility to sea lice by altering salmon conditions and/or stress levels, particularly by chemically and physically modifying mucus, possibly impacting its function of the innate immune barrier (Reverter et al., 2018).

This report details two experimental studies about the susceptibility of Atlantic salmon to the copepodids of *L. salmonis* following two experimental models of stressful events (e.g. handling, netting, pumping, transferring, crowding) that salmon can undergo during commercial salmon farming, and particularly during sea lice infestation monitoring/management procedures (such as lice counting and delousing treatments): (a) the effect of handling by netting and air exposure (model of acute stress); (b) the effect of crowding with restricted surface access in a shallow tank during several days (model of chronic stress). This was achieved by investigating the number of attached copepodids in association with some known physiological markers of stress (plasma cortisol and neutrophil to lymphocyte ratio; Davis et al., 2008; Sheriff et al., 2011).

## 2 MATERIALS AND METHODS

### 2.1 Experimental animals

This study was conducted under the approval of the Ethical Committee of IRSEA (no. 125) and in accordance with the European Directive 2010/63/UE for the protection of laboratory animals. Atlantic salmon were obtained on a farm (SALMAR®, Vikebukt, Norway) three weeks before the beginning of the experiments and were transferred directly from freshwater to sea water in the laboratory (i.e. smoltification). Smolts were kept in 180‐L holding tanks continuously supplied with filtered sea water (10 L/min, 10°C) pumped from Tresfjorden (62°32′25.5″N 7°08′18.1″E, Vikebukt, Norway). Salmon were reared under conditions of continuous light and fed ad libitum with commercial dry pellets (Skretting AS^©^, Norway). *L. salmonis* copepodids (Ilab^©^, Norway) were held in a net‐bucket system continuously supplied with filtered sea water (0.1 L/min, 10°C) and under continuous light.

### 2.2 Experiment 1: Susceptibility of handled salmon to *L. salmonis*

At T_0_, 64 Atlantic salmon smolts (94.1 ± 1.9 g) from the holding tank were introduced into 16 tanks (50 × 50 × 12 cm^3^) (four fish per tank). Each tank was continuously supplied with sea water (5 L/min, 10°C, continuous light). At T_1_, 20 hr after the transfer, four fish from eight tanks (“handled tanks”) were captured using a landing net and kept out from the water for 15 s. The fish from the eight other tanks (“control tanks”) were not handled.

Thus, 60 min after the handling procedure, the 16 fish from four tanks (two handled tanks and two control tanks) were transferred into a bath of lethal anaesthetic (0.7 ml/L Benzoak® from EuroPharma, Leknes, Norway) to assess their total plasma cortisol level. A blood sample was taken from the caudal vein of the fish using a 2.5 ml‐syringe and a 23G × 1″ needle. The blood was transferred to a 4 ml‐heparinized tube and stored in ice before centrifugation. Plasma was separated by centrifugation of blood samples (2,500 *g* for 15 min) at 4°C, removed and aliquoted into 1.5‐ml microcentrifuge tubes. Samples were stored at −18°C prior to subsequent analysis. Total plasma cortisol levels were determined using an enzyme‐linked immunosorbent assay (ELISA) kit (Enzo Life Sciences, Villeurbanne, France), as previously reported by Delfosse et al. (2016).

The in vivo test of *L. salmonis* attachment success on salmon was performed according to Delfosse et al., 2018, with slight changes due to the tank size. Briefly, 30 min after the handling procedure, 2,880 copepodids *L. salmonis* were introduced in the 12 remaining tanks (six handled and six controls, 240 copepodids per tank). Copepodids were accurately counted under binocular magnification by combining them in the wells of an ELISA plate using Pasteur pipettes. Then, 45 min after the introduction of copepodids, the 48 fish from the 12 infested tanks were transferred to a bath of lethal anaesthetic (0.7 ml/L Benzoak®). The fish were first visually examined to detect possible skin lesions, especially on head, fins, body. If such lesions were detected, the fish was excluded from the counting procedure. Three times, the entire body of each fish was scrubbed and rinsed above a plastic bag. The content of the bag was then filtered, and the plastic bag was again rinsed two times, and this liquid was also filtered, to count the number of copepodids attached to each fish using a magnifying glass, as previously validated (Delfosse et al., 2018).

### 2.3 Experiment 2: Susceptibility of crowded salmon with restricted surface access to *L. salmonis*

**FIGURE 1:**
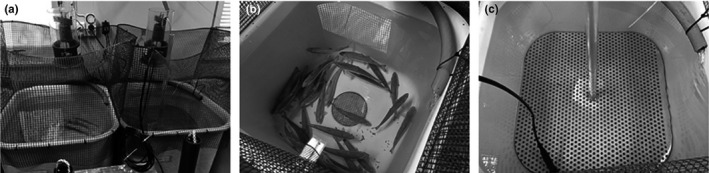
Pictures of the experimental system used in Experiment 2 to confine the Atlantic salmon smolts. (a) Side view of the two holding tanks: the control tank is on the left; the treated tank is on the right. (b) Top view of the control tank. (c) Top view of the treated tank, with the surface rigid metallic platform pierced with small holes on the top and pushing down the smolts hence increasing their stocking density and preventing surface access

Thirty‐two salmon smolts were added to two 180‐L holding tanks (16 smolts per tank) continuously supplied with filtered sea water (5 L/min, 10°C). The crowding procedure was performed in one of the two holding tanks. The 16 Atlantic salmon smolts from this tank were densely packed, and their surface access was limited by reducing the available water volume by one‐third and by continuously preventing them from rising to the surface (and filling their air bladder). This was performed using a surface rigid metallic platform pierced with small holes and pushed downward. The platform was removed, and the fish were allowed to reach the water surface for only 2 hr per day (randomly 1 hr in the morning and 1 hr in the afternoon). The system used is shown in Figure 1. This procedure was conducted for 5 days (from D_0_ to D_4_).

On D_5_, 8 smolts from each tank were removed and introduced in a lethal bath of anaesthetic (Benzoak®, 0.7 ml/L). A blood sample was taken from the caudal vein of each fish. A drop of blood was used to obtain a blood smear to measure the neutrophil:lymphocyte (N:L) ratio for each salmon; the remaining blood was injected into heparinized tubes and stored at 4°C before centrifugation to measure the total plasma cortisol level.

Plasma was separated by centrifugation (2,500 *g* for 12 min) at 4°C, removed, and aliquoted into 1.5‐ml microcentrifuge tubes. Samples were stored and assayed for total plasma cortisol levels as stated in experiment 1. Blood smears were stained with a commercial May‐Grunwald and Giemsa kit (RAL 555 KIT, RAL Diagnostics, Martillac, France), and each blood smear was read by two blinded operators. In each sample, a total of 100 cellules (lymphocytes and neutrophils) were counted. The N:L ratio was calculated by taking the mean values of the two operators’ measurements.

The salmon involved in the test of *L. salmonis* attachment success were different from the salmon used for blood samples, but they came from the same holding tanks. The test was performed as described in Delfosse et al. (2018) in individual flat beakers equipped with valves (3.5 L, Ø 23 cm) and supplied with sea water. Briefly, at D_5_, immediately after blood sampling, 8 Atlantic salmon smolts (137.8 ± 10.6 g) from each of the two holding tanks were placed into 16 flat beakers. Previously counted under a binocular microscope, 60 *L. salmonis* copepodids were injected into each flat beaker. Forty‐five min after the introduction of copepodids, 2 ml of anaesthetic (Benzoak®, lethal dose) was injected into each flat beaker. The fish were first visually examined to detect possible skin lesions, especially on head, fins, body. If such lesions were detected, the fish was excluded from the counting procedure. Three times, the entire body of each fish was scrubbed and rinsed above a plastic bag. The content of the bag was then filtered, and the plastic bag was again rinsed two times, and this liquid was also filtered, to count the number of copepodids attached to each fish using a magnifying glass, as previously validated (Delfosse et al., 2018).

### 2.4 Statistical analysis

Data were analysed using 9.4 SAS software (2002–2012 by SAS Institute Inc., Cary, NC, USA). The significance threshold was conventionally set at 5%.

For experiment 1, the experimental unit corresponded to the fish for the cortisol measurement and to the tank for the infestation test, the number of attached copepodids being dependent among fish in the same tank. The mean number of attached copepodids was computed for the four salmon contained in each tank for the outcome of attachment success, expressed in number of attached copepodid per fish. The comparison of plasma cortisol concentrations and number of attached copepodids between the handled and control groups was carried out using a Wilcoxon two‐sample test and *npar1way* procedure in SAS 9.4 software.

For experiment 2, all data were tested for evidence of departures from the assumption of normality using residual diagnostic plots and the *univariate* procedure in SAS. The assumption of homoscedasticity was verified with a Fisher test available in the *ttest* procedure of SAS. Comparisons were made between densely packed and control salmon for all the parameters measured using a Wilcoxon two‐sample test and *npar1way* procedure or using Student's *t* test and the *ttest* procedure according to normality and homoscedasticity.

## 3 RESULTS

### 3.1 Experiment 1

**FIGURE 2:**
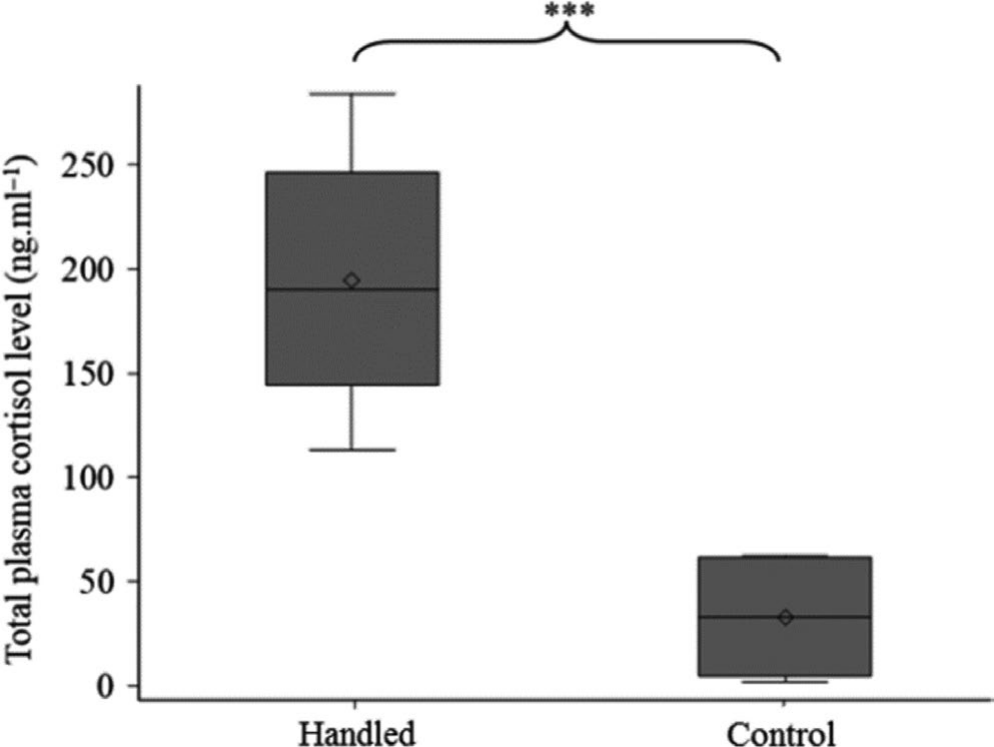
Plasma cortisol concentration in Experiment 1. Boxplot of total plasma cortisol concentration of Atlantic salmon subjected to the handling procedure and controls. (***) indicates statistically significant differences between the two groups (|Z| = 3.31, *p* < .001; *n* = 8)

**FIGURE 3:**
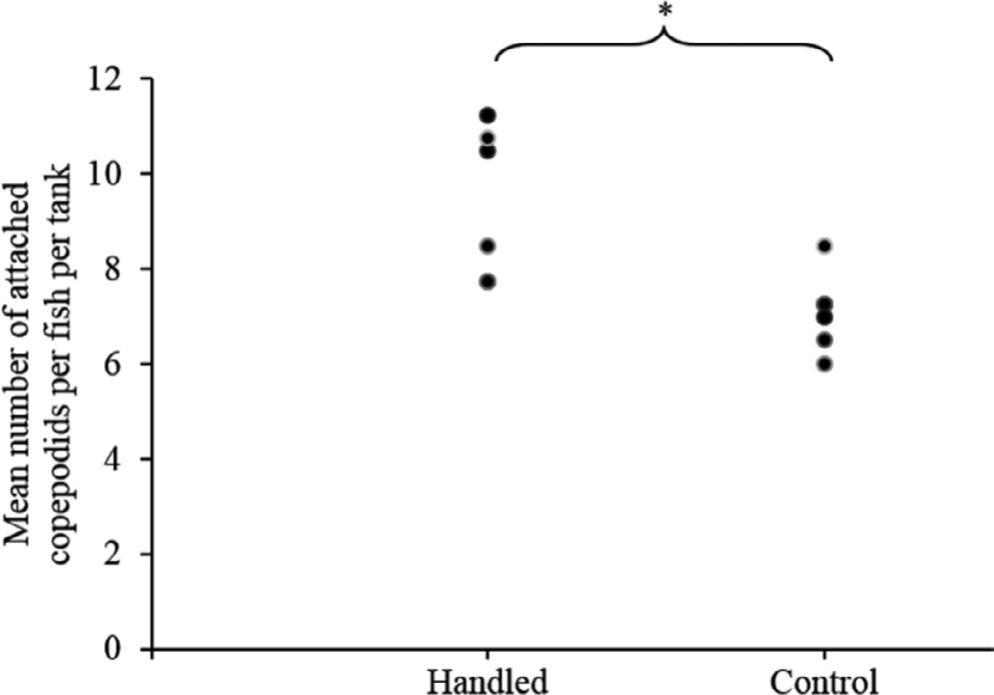
Number of attached copepodids in Experiment 1. Scatterplot of the mean number of attached *L. salmonis* copepodids per fish and per tank (*n* = 6 tanks per group) in the handled and control Atlantic salmon groups. (*) indicates statistically significant differences between the two groups (|*Z*| = 2.41; *p* = .02; *n* = 6)

The results from the total plasma cortisol levels and the number of attached copepodids obtained from the two treatment groups are reported in Figures 2 and 3, respectively. In the handled salmon (*n* = 8), the mean ± *SE* total plasma cortisol concentration was 195.6 ± 22.0 ng/ml compared to 33.2 ± 9.7 ng/ml for the control salmon (*n* = 8). The Wilcoxon two‐sample test showed that the total plasma cortisol concentration was significantly higher in the handled subjects (median = 190.7) than in the control subjects (median = 33.5; *p* < .001).

The mean ± *SE* number of attached copepodids per fish in the “handled tanks” (*n* = 6) was 9.4 ± 0.7, while it was 7.1 ± 0.4 in the “control tanks” (*n* = 6). The number of attached copepodids was thus statistically higher in the handled fish tanks (median = 9.5) than in the control tanks (median = 7.1; *p* = .01). No fish were excluded from the counting procedure due to possible skin lesions after visual examination.

### 3.2 Experiment 2

**FIGURE 4:**
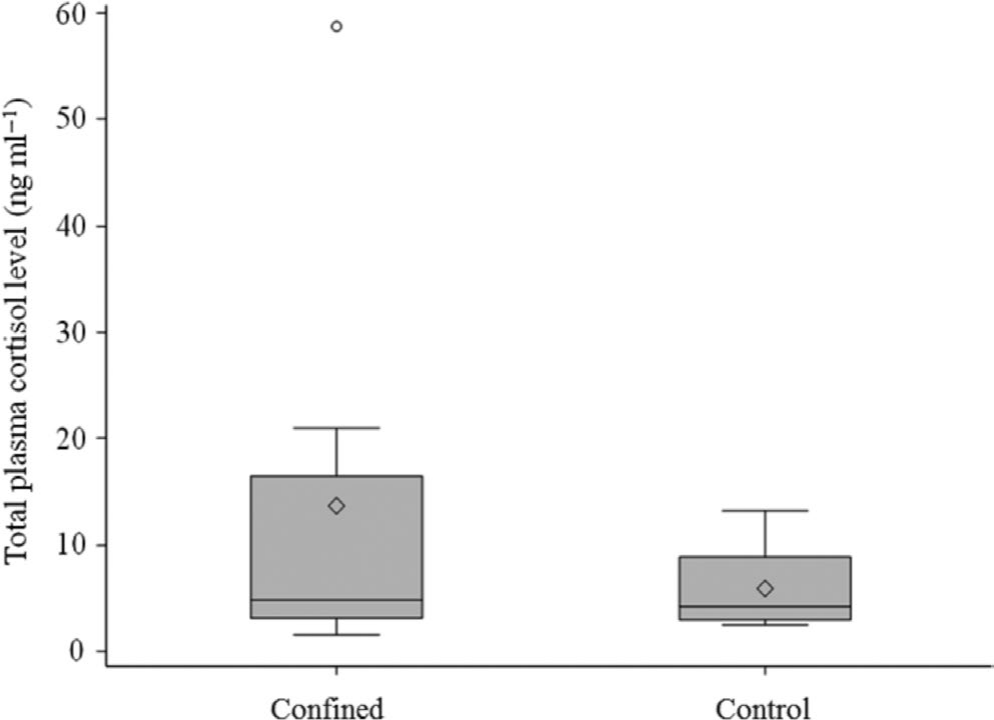
Plasma cortisol concentration in Experiment 2. Boxplot of total plasma cortisol concentration of confined Atlantic salmon and control subjects. No significant difference was observed between the two groups (|*Z*| = 0.3676; *p* = .71; *n* = 8)

Regarding the plasma cortisol concentration, the results of the comparison between the chronically densely packed and control salmon are shown in Figure 4. In densely packed salmon (*n* = 8), the mean ± *SE* total plasma cortisol concentration was 13.7 ± 6.8 ng/ml compared to 6.0 ± 1.5 ng/ml for the control group (*n* = 8). The Wilcoxon two‐sample test showed no significant difference in the total plasma cortisol concentration between the densely packed subjects (median = 4.9) and control subjects (median = 4.2; *p* = .71). The mean ± *SE* N:L ratio was 0.29 ± 0.03 for the densely packed salmon (*n* = 8) and 0.17 ± 0.01 for the control subjects (*n* = 8). The N:L ratio was significantly higher in the confined group than in the control group (median = 0.26 vs. 0.18; Wilcoxon two‐sample test; *Z* = 3.20; *p* = .0014).

**FIGURE 5:**
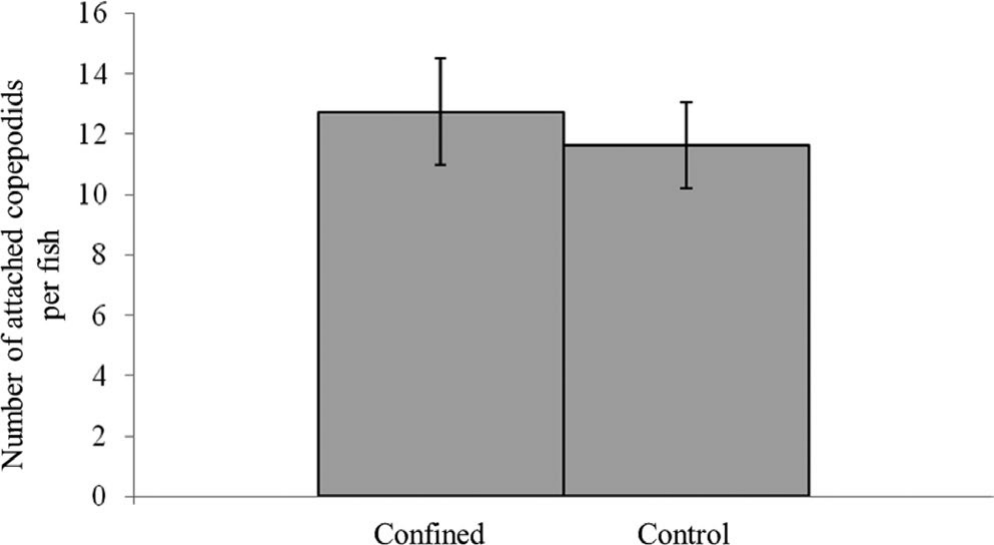
Number of attached copepodids in Experiment 2. Bar plot (mean ± *SE*) of the number of attached *L. salmonis* copepodids per fish in the confined and control Atlantic salmon groups. No significant difference was observed between the two groups (|*t*| = 0.49; *p* = .63; *n* = 8)

For the test of *L. salmonis* attachment success, the mean ± SE number of attached copepodids was 12.7 ± 1.8 for the densely packed salmon (*n* = 8) compared to 11.6 ± 1.4 for the control salmon (*n* = 8). Student's *t* test showed no significant difference between the two groups for the number of attached copepodids (*p* = .63; Figure 5). No fish were excluded from the counting procedure due to possible skin lesions after visual examination.

## 4 DISCUSSION

This study appears to be the first to examine the effect of handling and overcrowding on the susceptibility of Atlantic salmon to *L. salmonis* copepodids attachment under experimental conditions in a validated infestation test. The results showed that in comparison to the unhandled control salmon, the handled salmon presented a higher number of attached *L. salmonis* copepodids. In contrast, the number of attached copepods did not differ between the densely packed and control Atlantic salmon smolts in an individual infestation test. Interestingly, the procedures applied to the salmon also induced different outcomes regarding the plasma cortisol level; in comparison to the control salmon, the handled salmons presented higher total plasma cortisol levels 1 hr after the handling procedure, which could suggest an acute stress state, while the densely packed salmons did not present these levels. In fact, the plasma cortisol level of the densly packed salmon was found at basal levels after five days of overcrowding and surface access limitation procedures. Nevertheless, in comparison to the control salmon, the densely packed salmon presented a higher N:L ratio, indicative of a chronic stress state.

The handling procedure we used here was previously performed by Fast et al. (2008) to study the physiological response of Atlantic salmon after short‐term handling stress, and our results are in line with those found in the previous study on total plasma cortisol concentration. These authors also demonstrated an effect of short‐term and long‐term stress on immune cells. In addition, Gil Barcellos et al. (2004) observed similar effects on plasma cortisol and lymphopenia in male jundià (*Polycentrus jundia*, Coutinho et Wosiacki, Perciformes, Polycentridae) after acute and chronic stress, respectively, induced by classical aquaculture management procedures (handling and high density of fish in the tank, respectively). In salmon aquaculture, several classical procedures have also been shown to be detrimental to fish health status and welfare (Liu et al., 2015; Turnbull et al., 2005). For instance, handling during a vaccine injection can be a cause of stress, inducing immune suppression and thus being a limiting factor for vaccine efficacy (Sommerset et al., 2005). Sea lice treatments also have a substantial effect on salmon mortality and compromise fish welfare, regardless of whether there are chemotherapeutants or mechanical or thermal treatments (Overton et al., 2018).

As a complement to these findings and the known potential effect of some aquaculture practices on fish stress (Wendelaar Bonga, 1997), our results interestingly indicate a link between the plasma cortisol concentration and the *L. salmonis* copepodid infestation level. Notably, this effect seems to occur quickly since 30 min after the handling procedure is enough to observe changes in the number of attached copepodids in handled versus control fish. This outcome could be due to the depressive effect of cortisol on a number of immune responses in fish (Harris & Bird, 2000) that result in lowering salmon immunocompetence, including its innate immune defence; hence, this process allows a greater attachment of copepodids. However, the quickness of the observed effect does not favour a cortisol systemic action.

We propose that among the mechanisms driving handled salmon to be more susceptible to copepodids attachment, the elevation of total plasma cortisol may play a role by modifying the release and/or the composition of semiochemicals from salmon mucus, resulting in increased susceptibility. This scenario may support the idea that *L. salmonis* copepodids more readily attach to salmon because of this change in mucus composition and possibly the release/composition of semiochemicals. Núñez‐Acuña et al. (2018) recently showed that the antimicrobial peptide cathelicidin‐2 present in Atlantic salmon skin and mucus is a molecular host‐associated cue for the salmon louse *L. salmonis,* as it is detected by the parasite which can then modify its swimming behaviour. For instance, it could be of interest to study the effect of cortisol and/or stressing procedures on the production of this molecule in skin mucus to test our hypothesis. From an ecological perspective, host identification and host attachment are critical steps for ectoparasites with free‐living life stages, such as *L. salmonis* (Fields et al., 2018; Tucker et al., 2000b). Therefore, it might be possible that a copepodid is more attracted to and swims towards a stressed salmon, which could be a less immunocompetent fish, i.e. a most suitable host, to maximize its chances of attaching to salmon skin by developing its frontal filament; then, the copepodid could settle on it and complete its life cycle (Mordue & Birkett, 2009).

Alternatively, another factor causing handled salmons to be more susceptible to copepodids attachment than densely packed salmon might be abrasions due to netting. Handling procedures may induce physical injuries to the skin, loss of scales and mucus in salmon. Thus, ectoparasites could be better attached to handled salmon because of this mechanical deterioration of the natural epithelial/mucosal barriers, which are an important part of the innate immune system (Magnadottir, 2010). Besides the physical barrier function that skin and mucus stand for to protect the fish against parasites, mucus plays a key role in the first defence line also because of its content in numerous innate components such as enzymes (protease, esterase, lysozyme), antimicrobial peptides, complement proteins, heat‐shock proteins, immunoglobulins… (Koshio, 2016). Specifically, these molecular innate immune components of mucus seem to interact with *L. salmonis* infestation success in Atlantic salmon: indeed, Holm et al. (2015) suggest that the ability to resist lice infection depends on the ability to avoid immunosuppression (Holm et al., 2015). In both experiments of our study, based on a visual inspection, we did not notice any macroscopic skin lesions on the head, fins, body or scales loss. However, this approach did not allow us to assess the existence of some microscopic skin lesions or mucus alterations, and consequently, we cannot exclude these were present. Because of the importance of mucus for lice resistance, in further studies, it may be of interest to analyse the mucus composition and quantity/thickness and assess the quality of the scale and skin appearance on a microscopic level in handled and unhandled smolts to verify these hypotheses. Interestingly, Easy & Ross (2010) previously showed that handled salmon with relatively higher plasma cortisol levels presented changes in mucus composition, especially in mucus proteins, because of an increase in protease activity.

In contrast, in experiment 2, compared to the control smolts, densely packed smolts did not present any difference in plasma cortisol levels and can be considered at basal levels (<15 ng/ml (Martinez‐Porchas et al., 2009)). The smaller stress effect observed for crowding than handling may be explained given that farmed salmon often form dense schools voluntarily, resulting in increased fish densities in some areas of the cage (Oppedal et al., 2011). Conversely, netting and air exposure are among the most stressful events that can happen to a fish, regardless of the physical injury from netting (Nguyen et al., 2014). There was also no difference between densely packed and control salmons for the number of attached copepodids, thus reinforcing the hypothesis of a cortisol influence on the susceptibility of salmon to *L. salmonis* copepodids.

However, in comparison to the control salmon, the densely packed salmon presented a higher N:L ratio. The reduction of blood lymphocytes during chronic stress is well known in fish (Barton et al., 1987; Espelid et al., 1996; Frazer, 2009; Gil Barcellos et al., 2004; Whelan, 2010). In fact, circulating glucocorticoids are directly associated with an increase in neutrophils and a reduction in lymphocytes in blood (Davis et al., 2008), resulting in an increase in the N:L ratio. As explained by these authors, this neutrophilia associated with lymphopenia during stress is mainly due to a redistribution of lymphocytes and neutrophils in different body compartments. Compared to the plasma cortisol level, which diminishes over time, the leucocyte response to stress lasts longer and is more enduring, thus making the N:L ratio a reliable indicator of long‐term stress (Davis et al., 2008; Goessling et al., 2015). These results may suggest that the salmon which underwent crowding and restricted surface access in our study was in a long‐term stress state, and the plasma cortisol being at a basal level may reinforce this suggestion. Indeed, in the case of chronic stress, many studies in fish have demonstrated that plasma cortisol levels return to the basal level after a few days due to a decrease in the ACTH sensitivity of the interrenal tissue or the habituation of the organism to that condition (Gil Barcellos et al., 2004; Martinez‐Porchas et al., 2009; Mommsen et al., 1999). In particular, in Atlantic salmon, Madaro et al. (2015) showed in another model of chronic stress based on repeated exposure to unpredictable stressors for several days that plasma cortisol decreases steadily over time in stressed fish, indicative of exhaustion of the endocrine stress axis; after 5 days, cortisol levels in the stressed group were not different compared with those in the control group. This result is in line with the results we obtained in our model of long‐term stress after 5 days of restricted access to surface/overcrowding (except for 2 hr per day). In contrast, shorter durations (30 min to 6 hr) of confinement have been shown to elevate plasma cortisol levels and are considered acute stress conditions (Mes et al., 2018; Sadler et al., 2000). The increase of N:L ratio, indicative of long‐term stress state, observed in experiment 2 may be the result of the increased stocking density in the tank or the surface access restriction or both these factors together. These results based on haematological parameters are in line with previously published data about the fish condition and other stress parameters on the negative welfare outcomes of high stocking density; however, we should note that the stocking density applied in our procedure (approximately 18.4 kg/m^3^ at the end of the experiment, except during the two hours when the platform was removed and the stocking density was 12.3 kg/m^3^) is inferior to the threshold previously described as jeopardizing salmon welfare (Liu et al., 2015; Turnbull et al., 2005). Nevertheless, they are still comparable to the final stocking densities reached in commercial snorkel sea cages, estimated from the data published by Wright et al., 2017 (considering a 40‐times increase of the biomass at the end of the production cycle). In addition, it is has been shown that short‐term and long‐term submergence trials in commercial sea cages (in which the salmon cannot access to the surface) could also negatively impact the welfare of Atlantic salmon (Dempster et al., 2008; Korsøen et al., 2009). Then, further studies would be necessary to determine whether the chronic stress state is caused by the crowding or the limited surface access or both.

Regarding lymphopenia, the reduction could lead to a decreased immune response to environmental pathogens. In this study, the mean number of attached copepodids was the same between lymphopenic salmon and control subjects. These data do not allow us to clarify the role of immunity status on the first stages of the infestation behaviour of copepodids: recognition and attachment. To further develop our understanding of the role of immunity, it may be of interest to assess immune system function in the mucus and skin of salmon by measuring IgM antibodies or evaluating immune cell distribution (Hatten et al., 2001; Tadiso et al., 2011).

In the present study, all the smolts came directly from freshwater and were transferred and reared in filtered sea water in the laboratory to avoid external infestation by *L. salmonis*. The mean number of attached copepodids per fish in both experiment 1 and 2 (approximately 12 and 21% of introduced copepodids, respectively) can be considered to be consistent with other studies carried out under these conditions (water temperature, 10°C) (Delfosse et al., 2018; Delfosse & Pageat, 2014; Pageat & Delfosse, 2014).

Regarding the parasitic pressure in our studies (between 8,000 and 18,000 cop./m^3^), the number of copepodids was very high compared to what we find in salmon farms (between 0.02 and 0.3 cop./m^3^; á Nordi et al., 2015; Penston et al., 2008). This high parasitic pressure was based on previous studies (Browman et al., 2004; Tucker et al., 2000a) to ensure adaptation to laboratory conditions. This high number of copepodids allowed us to obtain enough variability in the data to highlight the putative effect of handling and overcrowding/surface access limitation on the susceptibility of the salmon to the copepodids.

## 5 CONCLUSION

Our experiments investigated the effect of handling and overcrowding combined with a restricted access to the surface on the attachment of *L. salmonis* copepodids to Atlantic salmon, suggesting a role of fish stress state in this interaction. Long‐term overcrowding had no effect on *L. salmonis* attachment and did not influence the first stage of the infestation behaviour of the sea louse: recognition and attachment. Nevertheless, overcrowding and restricted access to the surface were shown to increase the N:L ratio of Atlantic salmon, indicative of a long‐term stress state in these salmon, thus possibly raising concern about fish welfare after aquaculture procedures that could limit surface access and intensify local fish crowding. Importantly, this study shows that handling procedures influence the susceptibility of Atlantic salmon to *L. salmonis* copepodids, also proposing an increase in plasma cortisol as one possible aetiology. Many of the current lice management strategies may, paradoxically, increase reinfestation risk by handling or otherwise stressing the salmon. As recently stated by Overton et al. (2019), “the push for effective lice control and preventive strategies that have acceptable fish welfare outcomes are more important than ever,” emphasizing the need for a holistic approach to manage sea lice infestation in Atlantic salmon aquaculture.

## CONFLICT OF INTEREST

The authors have no conflict of interest to declare.

## Data Availability

Here is no shared data.
